# Salidroside inhibits MAPK, NF-κB, and STAT3 pathways in psoriasis-associated oxidative stress via SIRT1 activation

**DOI:** 10.1080/13510002.2019.1658377

**Published:** 2019-09-07

**Authors:** Fengli Xu, Jixiang Xu, Xia Xiong, Yongqiong Deng

**Affiliations:** Department of Dermatology, The First Affiliated Hospital of Southwest Medical University, Luzhou, People’s Republic of China

**Keywords:** Psoriasis, oxidative stress, SIRT1, MAPK, NF-κB, salidroside, ROS, STAT3

## Abstract

**Objectives:** To unveil the role of SIRT1 in limiting oxidative stress in psoriasis and to further discuss the therapeutic prospects of salidroside in psoriasis.

**Methods:** Literature from 2002 to 2019 was searched with “psoriasis”, “oxidative stress”, “SIRT1”, “salidroside” as the key words. Then, Oxidative stress in psoriasis and the role of SIRT1 were summarized and the potential role of salidroside in the disease was speculated.

**Results:** Oxidative stress might contribute to the pathogenesis of psoriasis. High levels of ROS produced during oxidative stress lead to the release of inflammatory mediators, that, in turn, induce angiogenesis and excessive proliferation of keratinocytes. SIRT1 is a member of the sirtuin family, of which the activation lead to the inhibition of such oxidative stress signaling pathways MAPK, NF-κB, and STAT3, down-regulation of inflammatory factors, suppression of inflammation and keratinocyte hyperproliferation, and inhibition of angiogenesis. Salidroside, the main ingredient of Rhodiola, is known to exert antioxidant roles, which has been attributed to SIRT1 activation.

**Conclusion:** Salidroside might inhibit oxidative stress singling pathways via SIRT1 activation, and could be as an ideal candidate for management of psoriasis.

## Introduction

Psoriasis is a chronic inflammatory disease that affects 2%–5% of the world’s population. It is characterized by erythema, plaques, and scales, and is sometimes accompanied by itching [1]. The histological hallmarks of psoriasis include angiogenesis, abnormal keratinocyte proliferation, and infiltration of inflammatory cells. Some investigators have highlighted the genetic factors involved in the pathogenesis of psoriasis. For example, TNF-*α* gene polymorphisms, including the SNP +489 allele, have been significantly associated with psoriatic arthritis susceptibility and severity [2]. Moreover, other previous studies have established their association with autoimmune diseases and environmental factors. Recently, it has been reported that the generation of oxidative stress could promote psoriasis through the MAPK, NF-κB, and STAT3 pathways and that it can be alleviated by inhibition of these three pathways [3,4].

Salidroside – the main ingredient of Rhodiola – has anti-aging and antioxidant properties. Recent reports have emphasized its protective effects on the nervous and cardiovascular systems, and in the treatment of tumors and inflammatory diseases [5]. Salidroside reportedly plays an antioxidant role via activation of SIRT1, which could serve as a new target for treating psoriasis. This review aims to elucidate the role of SIRT1 in mediating oxidative stress in psoriasis and to discuss the potential therapeutic role of salidroside in psoriasis.

## Oxidative stress in psoriasis and the role of SIRT1

Oxidative stress is generated due to an imbalance between levels of reactive oxygen species (ROS) and antioxidants [6]. ROS mainly include superoxide anion (O_2_^−^), hydroxyl radical (HO^−^), hydrogen peroxide (H_2_O_2_), and lipid radicals, which are the byproducts of cellular metabolism [7]. Some enzymes, such as superoxide dismutase (SOD), catalase (CAT), and glutathione peroxidases (GSH-px), function as antioxidants, protecting cells from oxidative damage [8].

Oxidative stress is an essential factor that induces and aggravates psoriasis, which is known to be induced by a variety of factors – alcohol consumption, smoking, infection, drugs, obesity, cell metabolism, immune response, and pathological state [9]. Previous reports showed that the total antioxidant capacity (TAC) in patients with psoriasis is lower than that in healthy subjects [10–12]. Although severe oxidative stress leads directly to cell death, recent research has demonstrated that mild oxidative stress plays a more critical role in the pathogenesis of psoriasis than severe oxidative stress [4].

Generation of ROS is a crucial step in the induction of oxidative stress in psoriasis. ROS generally act as second messengers during this process and lead to an increase in the levels of malondialdehyde (MDA), NO, HO^−^, and inducible nitric oxide synthase (iNOS), and decrease in the levels of SOD, CAT, and GSH-px [3,9,13]. Elevated levels of oxidative products result in the activation of Th1 and Th17 cells and keratinocytes through the MAPK, NF-κB, and JAK-STAT pathways [12,14]. This results in a cascade of inflammatory cytokines and growth factors, such as IFN-*γ*, IL-2, TNF-*α*, and TNF-β, produced by Th11; IL-17, IL-22, IL-23, and TNF-*α*, by Th17; and antimicrobial peptide (AMP), TNF-*α*, IL-6, IL-8, polymorphonuclears (PMN), and VGEF, by activated keratinocytes [12,15]. These inflammatory mediators further activate T cells and mast cells to give rise to a self-amplifying loop that results in keratinocyte overproliferation, neutrophil recruitment, hypervascular hyperplasia, and sustained skin inflammation [15,16]. Angiogenesis – a marker of psoriasis pathogenesis – could also be promoted by angiogenic mediators like VEGF, TNF-*α*, IL-8, and IL-17 [16,17]. As reported, oxidative stress and a high level of TNF-*α* induce endothelial dysfunction, which can consequently contribute to autoimmune and cardiovascular diseases [18,19].

Sirtuins are a family of proteins that are involved in multiple cellular functions related to metabolism, cell cycle, aging, inflammation, apoptosis, cell proliferation, and DNA repair [20–22]. Among them, SIRT1 is the most well studied. SIRT1 is a highly conserved NAD^+^-dependent histone deacetylase that mediates the antioxidative stress in psoriasis [23,24]. It was reported that SIRT1 could promote the differentiation of human keratinocytes [25] and inhibit keratinocyte proliferation [26]. Moreover, SIRT1 protects fibroblasts of individuals with psoriasis from oxidative stress-induced apoptosis and restores both mitochondrial function and redox balance via downregulation of MAPK signaling [12]. Additionally, Wang Y et al. reported that chemerin exacerbated psoriasiform dermatitis in a mouse model and that it can induce inflammatory response and promote NF-κB activation by inhibiting the activity of ROS-induced SIRT1 [27]. Recently, a study found that the expression levels of SIRT1-5 were downregulated, while those of SIRT6 and SIRT7 were upregulated in psoriasis skin lesions [28]. According to the literature, many compounds, such as dioscin, grape seed oil, luteolin, resveratrol, methyl ferulic acid, and STR1720, can activate or/and upregulate SIRT1 [29].

## SIRT1/MAPK/AP-1 signaling cascade

MAPKs are a family of serine-threonine protein kinases that are involved in signal transduction, cell differentiation, proliferation, apoptosis, and immune response. Members of this family include – extracellular signal-regulated kinases (ERKs), c-Jun N-terminal kinases (JNKs), and the p38 MAPKs [4,30]. AP-1 is an important eukaryotic transcription factor that is activated by the three MAPK pathways and regulates various inflammatory factors such as TNF-*α*, IL-6, and MCP-1ex. The involvement of the MAPK/AP-1 pathway in oxidative stress has been extensively investigated [31–34]. It has been demonstrated that there is increased activation of ERK1/2, p38 MAPK, and JNK in lesional psoriatic skin [35–37], and JNK is involved in the differentiation and proliferation of keratinocytes [38]. Reports suggest that activation of SIRT1 protects the heart from oxidative stress and cushions the brain from alcohol-induced neurodegeneration via inhibition of the p38 MAPK pathway [39,40]. Studies indicate that carnosic acid protects normal mouse hepatocytes against oxidative stress-induced cytotoxicity by regulating ERK1/2 via SIRT1 [41]. When SRT2014, an agonist of SIRT1, was used to treat moderate to severe psoriasis, it was observed that the expression of known IL-17 and TNF-*α* responsive genes and that of genes involved in keratinocyte differentiation were significantly downregulated [42].

## SIRT1/NF-κB relationship

Nuclear factor-κB (NF-κB) is an essential inflammatory mediator in the pathogenesis of psoriasis; increased expression of NF-κB has been demonstrated in psoriatic lesions [4]. ROS can activate NF-κB through the phosphorylation of the inhibitor of kappa B kinase (IKK) complex [43,44]. Studies indicate that H_2_O_2_, imported via AQP3, participates in the activation of the NF-κB signaling pathway in keratinocytes and is involved in the pathogenesis of psoriasis [45]. Altered NF-κB signaling disrupts the balance of apoptotic signals, leading to the upregulation of cyclins and survivins, thereby inhibiting apoptosis [44]. Furthermore, NF-κB induces the production of IL-17 and TNF-*α*, thereby enhancing the downstream inflammatory response [44,46]. SIRT1 has been shown to suppress NF-κB signaling through deacetylation of the p65 subunit of NF-κB and resulting in the reduction of inflammatory responses [47]; overexpression of SIRT1 reduced the expression of IL-1β, IL-8, and TNF- *α* [27]. It has been recognized that SIRT1 can inhibit NF-κB signaling through AMPK, PGC-1*α*, and PPAR*α*; concomitantly, NF-κB also downregulated SIRT1 expression and signaling via ROS, IFN-*γ*, and PARP-1 under oxidative stress conditions [48].

## SIRT1/STAT3 interaction

Besides MAPK/AP-1 and NF-κB pathways, STAT3 also plays an essential role in psoriasis. STAT3 has been implicated in the regulation of fundamental biological processes such as cell proliferation, differentiation, oncogenesis, survival, and apoptosis [49]. In resting cells, STAT3 is localized in the cytoplasm. When stimulated by ROS, STAT3 gets activated through phosphorylation at Tyrosine 705 (Tyr705), following which it enters the nucleus and regulates gene expression. STAT3 is mainly phosphorylated by JAK and the epidermal growth factor receptor kinase; however, Src and ERK may also be involved in STAT3 phosphorylation [50]. In addition, MAPK, ERK, and protein kinase have been demonstrated to enhance STAT3 gene transcription [50,51]. Thus, MAPK and STAT3 synergistically promote the development of psoriasis. A variety of studies have demonstrated that STAT3 is over-active in psoriatic lesions, where it promotes the excessive proliferation of keratinocytes and the production of IL-6, IL-8, IL-23, and IL-17. These cytokines, in turn, trigger the Th17 and STAT3 signaling pathway, thereby resulting in continuous inflammation [52]. Ki67, keratin16, and keratin17 are markers of excessive proliferation of keratinocytes. Bin Zhang et al. (2016), reported that compared to that in healthy skin, the activity of STAT3 is high in psoriatic lesions, and there is an excessive proliferation of keratinocytes as is evidenced by high levels of Ki67, keratin16, and keratin17 staining. They also reported that stimulation with IL-6 and IL-22 results in increased phosphorylation of STAT3. In parallel, the downstream genes of STAT3 such as survivin, cyclin D1, and the Bcl family were upregulated [53]. There is compelling evidence that SIRT1 counteracts the IL-22-induced STAT3 activity by deacetylating STAT3 and represses the downstream gene expression, thereby inhibiting keratinocyte proliferation and migration [54]. Besides, SIRT1 has been shown to reduce the severity of lesions in the Aldara-induced psoriasis model via negatively regulating STAT3 activation. Moreover, a significant reduction in SIRT1 levels and conversely, an increase in PY-STAT3 were detected in psoriatic mouse models as compared with that in healthy mice [55].

**Figure 1.**
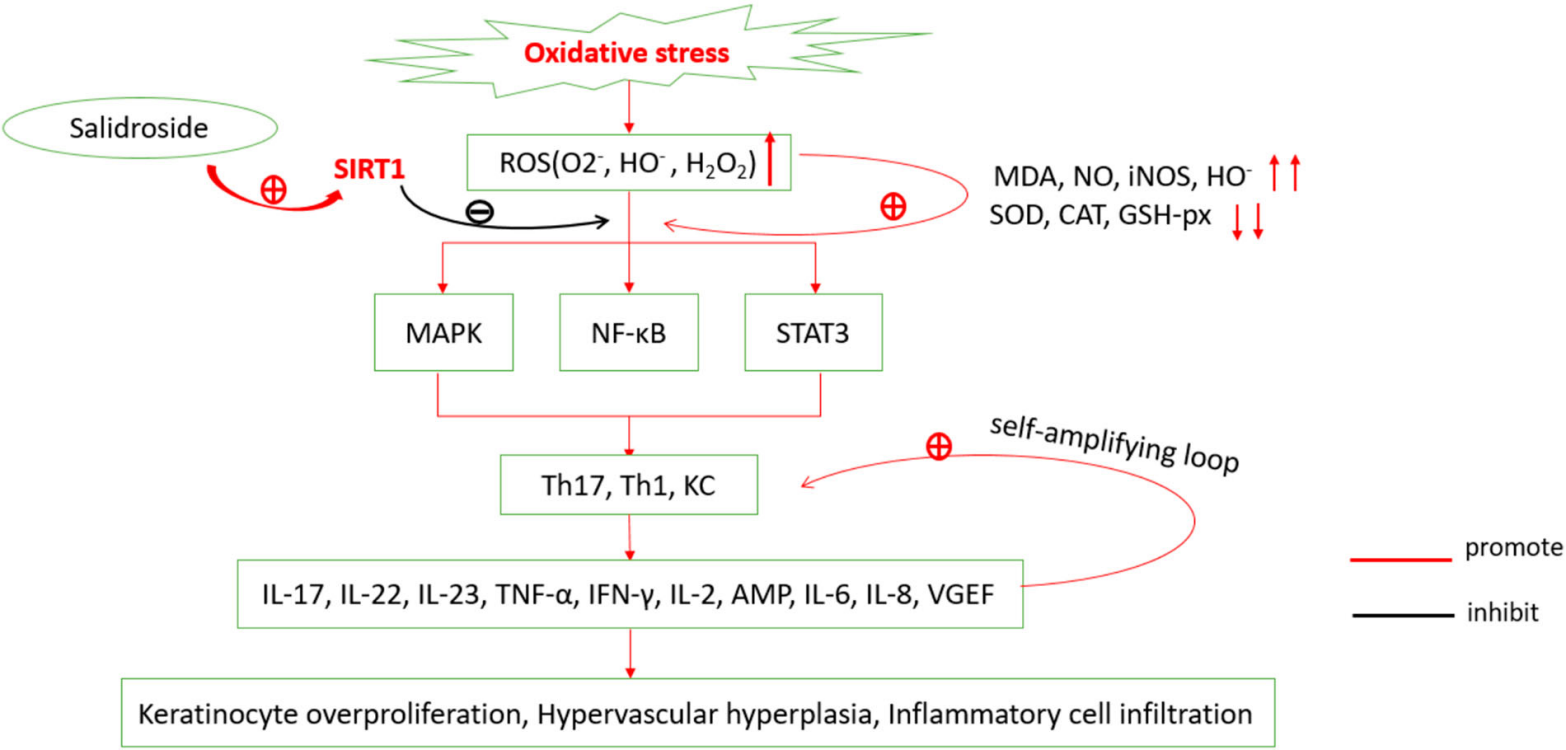
Psoriatic oxidative stress mechanism. Reactive oxygen species (ROS) including superoxide anion (O_2_^−^), hydroxyl radical (HO^−^), hydrogen peroxide (H_2_O_2_) are increased in oxidative stress in psoriasis, resulting in the increase of malondialdehyde (MDA), NO, HO^−^ and inducible nitric oxide synthase (iNOS); on the contrary, levels of SOD, CAT and GSH-px are decreased. The increased oxidative products lead to the activation of Th1, Th17, and keratinocyte cells through MAPK, NF-κB, JAK-STAT pathways, resulting in overproduction of IL-17, IL-22, IL-23, TNF-*α*, IFN-*γ*, IL-2, AMP, IL-6, IL-8, and VGEF. These inflammatory factors further activate Th1, Th17, and keratinocytes, forming a self-amplifying loop, eventually leading to keratinocyte overproliferation, hypervascular hyperplasia, and inflammatory cell infiltration. Salidroside, the activator of SIRT1, resists oxidative stress by inhibiting MAPK, NF-κB, and STAT3 pathways.

In summary, activation of SIRT1 can counteract the damage caused by oxidative stress by inhibiting the MAPK, NF-κB, and STAT3 pathways (Figure 1), leading to the alleviation of the pathological injury in psoriasis. Therefore, SIRT1 can serve as a new therapeutic target for this disease. However, to date, most studies have focused on antioxidants such as dimethyl fumarate, curcumin, and propylthiouracil for treating psoriasis [56–58]. Besides, studies using specific antibodies for the treatment of psoriasis have confirmed that biological agents are indeed effective against this condition [59,60]. However, these medicines have limited clinical applications due to adverse reactions, high cost, and inaccurate long-term efficacy. Thus, it is vital to develop a cheap and effective treatment with limited side effects for psoriasis.

## Salidroside

Recently, a detailed review of the pharmacological effects of salidroside in various diseases has demonstrated its anti-aging, anti-inflammatory, antioxidation, anti-cancer, and liver protection properties [61]. It is also non-toxic to astrocytes [62], improves hind limb motor function and reduces tissue damage in spinal cord injury by reducing the phosphorylation of NF-κB, ERK, and p38 and by reducing the production of inflammatory cytokines such as IL-1β, IL-6, and TNF-*α* [62]. Previous studies showed that salidroside can prevent DMBA/TPA-induced skin cancer in mouse models by inhibiting inflammation and promoting apoptosis [63]. Xiang-jun Fan et al. indicated that salidroside was able to inhibit the growth of HT-29 human colorectal cancer cells and induce cell apoptosis, possibly via the PI3 K/Akt/mTOR signaling pathway [64]. Studies have also shown that salidroside protected retinal pigment epithelial cells from oxidative stress induced by H_2_O_2_ [65]. Besides, salidroside reduces the production of LPS-induced proinflammatory cytokines and mediators and attenuates LPS-induced acute lung injury by inhibiting the JAK2-STAT3 signaling pathway [66].

In addition, salidroside could act as an activator of SIRT1 to exert its antioxidant effects. Chun-Yang Wang et al. proved that salidroside increased SOD activity and GSH levels and reduced the phosphorylation of MAPK by activating SIRT1; they also found that it protects SH-SY5Y cells from apoptosis and oxidative stress induced by MPP^+^ [67]. Similarly, salidroside protects against kainic acid-induced status epilepticus through suppression of oxidative stress, and this effect might be mediated by the AMPK/SIRT1/FoxO1 pathway [68]. It has also been reported to protect human umbilical vein endothelial cells (HUVECs) from oxidative stress induced by ox-LDL through increasing SIRT1, FoxO1, and SOD expression and to simultaneously down-regulate MDA and LDH [69]. Meanwhile, another research concluded that salidroside protects HUVECs against ox-LDL injury through inhibiting oxidative stress and improving mitochondrial dysfunction, which were dependent on AMPK/SIRT1 pathway activation [70]. Furthermore, Xu N et al. concluded that salidroside decreased the expression of inflammatory cytokines and improved LPS-induced learning memory impairments, which were involved in the SIRT1-dependent Nrf-2/HO-1/NF-κB pathway [71].

However, the antioxidative mechanism of salidroside in psoriasis has not yet been reported. In our review, we highlight the role of SIRT1 in reducing oxidative stress in psoriasis. The activation of SIRT1 could effectively inhibit MAPK, NF-κB, and STAT3 phosphorylation, thereby decreasing the expression of downstream genes and secretion of inflammatory cytokines, eventually blocking the development of psoriasis. Salidroside, the activator of SIRT1, may cure psoriasis resistant to oxidative stress damage by inhibiting the MAPK, NF-κB, and STAT3 pathways; however, this property of salidroside needs further research.

## Conclusion

This review, overall, indicates that psoriasis is a common dermatitis associated with oxidative stress mediated by MAPK, NF-κB, and STAT3 signaling cascade. Salidroside, the activator of SIRT1, may cure psoriasis by blocking these three targets. Thus, salidroside, alone or in combination with other compounds, could be an ideal candidate for the management. However, to date, no research on salidroside as a therapeutic option to treat psoriasis has been reported. Hence, further investigation is required to validate the effects of salidroside on psoriasis pathophysiology and further verify the effect of SIRT1 on MAPK, NF-κB, and STAT3 pathways in the skin.
